# Manufacturing Anti-Reflective Subwavelength Structures on ZnS Using Femtosecond Laser Bessel Beam with Burst Mode

**DOI:** 10.3390/biomimetics9110655

**Published:** 2024-10-26

**Authors:** Haoran Wang, Biwen Li, Liangbin Hu, Fan Zhang

**Affiliations:** 1 Key Laboratory of Hunan Province of Equipment Safety Service Technology Under Extreme Environment, University of South China, Hengyang 421001, China; 2 State Key Laboratory of Precision Manufacturing for Extreme Service Performance, Central South University, Changsha 410083, China; 3 College of Mechanical Engineering, University of South China, Hengyang 421001, China

**Keywords:** femtosecond laser, anti-reflective subwavelength structure, Bessel beams, burst mode

## Abstract

Increasing the transmittance of zinc sulfide (ZnS) infrared windows can effectively improve the imaging quality of infrared detection. In this study, an anti-reflective subwavelength structure (ASS) was manufactured on ZnS using a femtosecond burst Bessel laser with the goal of achieving high transmittance in the mid-infrared range. The period and depth parameters of the ASS were initially determined using the effective medium approximation (EMA) theory and subsequently optimized using the rigorous coupled-wave analysis (RCWA) method to eliminate surface Fresnel anti-reflections. The depth of the ASS increases with the number of bursts, while the structure profile transitions from Gaussian to conical. In addition, the ASS achieves 86% transmittance in the 7–10 µm range, and the average transmittance improves by 10% in the 5–12 µm range. Moreover, the wide-angle ASS with the hydrophobicity (contact angle 160°) is achieved on the ZnS window. Ultimately, the ASS on ZnS enhances the clarity of the infrared image.

## 1. Introduction

The infrared window is crucial in infrared detection [1], which is an important guarantee for infrared detection equipment to accurately collect infrared signals in national defense and military [2], future city construction [3], and industrial modernization [4]. Zinc sulfide has good infrared optical properties and is often used as an infrared window material for various detectors [5]. However, in the field of military defense, it is difficult for a single window material to meet the increasingly stringent infrared detection requirements due to the extreme operating conditions of the application environment or the influence of interfering factors in the environment [6,7,8,9]. Although a conventional anti-reflection thin-film coating (ARC) [10,11] can effectively improve the optical properties of zinc sulfide surfaces, it is still a technological challenge to design a coating that can be used in the entire mid-infrared spectral range (2–16 μm) [12,13,14]. In addition, it is also susceptible to thermo-mechanical damage and may even lead to major structural failures when subjected to laser or mechanical and chemical damage [15,16]. Anti-reflective subwavelength structures are typically sub-micron and micron in size, and when fabricated on the surfaces of infrared windows, they have similar optical properties to ARC [17,18]. Specifically, they can overcome ARC defects and enhance the fogging and damage resistance of infrared windows [19,20,21].

Femtosecond lasers have ultra-intense, ultra-fast, and ultra-precise properties, and the process of fabricating microstructures is simple and flexible, with maskless lasers being one example [22,23,24,25,26]. Furthermore, it is a method for processing ASS, which can effectively improve window transmittance for infrared detection applications [27]. For example, Liu et al. fabricated biomimetic micro/nanostructures on sapphire by femtosecond laser deep-scribing technology with the etching process, achieving 98% transmittance at 4 μm [28]. Teslenko et al. used a single-pulse femtosecond laser to fabricate anti-reflection structures on the surface of a LiGaSe_2_ crystal, achieving an average transmittance of 97.2% in the 2–8 μm range [29]. Ding et al. fabricated ASS on the surface of MgF_2_ using a femtosecond Bessel beam, achieving 99% transmittance in the broadband of 3–5 μm [30]. Teslenko et al. used femtosecond laser ablation and wet chemical etching to fabricate high-aspect-ratio anti-reflection microstructures on ZnSe surfaces, improving transmittance in the mid- and far-infrared regions [31]. In the face of extremely complex environments and diverse infrared detection needs, the development of an efficient femtosecond laser processing technology with broad wavelength bands and high transmittance remains a great challenge [17].

In this paper, anti-reflective subwavelength structures are fabricated on ZnS infrared windows using a spatial shaping femtosecond burst Bessel laser to suppress surface Fresnel reflections. The structural parameters of the anti-reflective subwavelength structures are determined using RCWA and adjusted based on laser processing parameters. Additionally, spectral transmittance, wettability, and infrared imaging tests are conducted to evaluate and enhance the functionality of the ASS on the ZnS surface.

## 2. Materials and Methods

In this paper, the primary period and depth were calculated by using EMA. Then, RCWA was used to optimize the ASS parameters for ZnS surfaces. The Z direction was designated as the beam incidence direction, while the X direction and Y direction were defined by the transmission-line treatment and periodic boundary conditions. To simulate the electric field intensity distribution of microstructures in the 2.5–13.5 um range, a 10 nm grid was set. Additionally, to ensure stable transmittances, the simulation time was set to 5000 fs.

**Figure 1:**
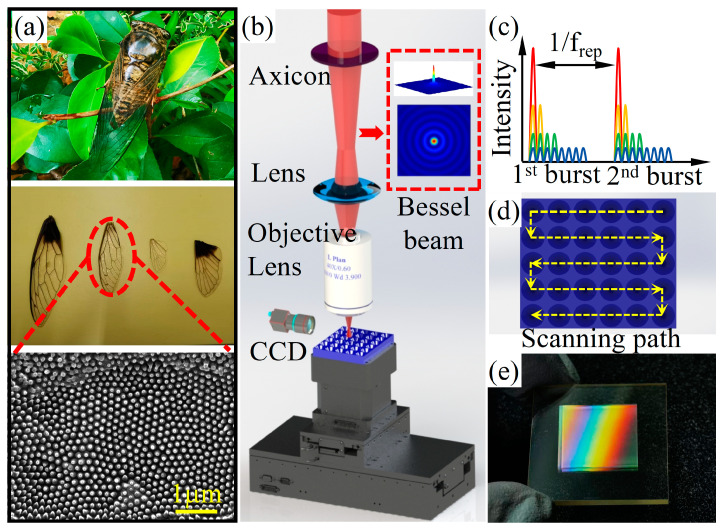
(**a**) Photograph of cicada wings and SEM image of transparent anti-reflective area; (**b**,**c**) schematic diagram of femtosecond burst Bessel processing system (modes consisting of N = 1, 2, 4, and 8 pulses with t_sep_ = 25 ns); (**d**) schematic diagram of manufacturing process of conical array zinc sulfide anti-reflective surface; (**e**) image of ZnS anti-reflective surface under light.

As shown in Figure 1b, a Yb: KGW Gaussian beam femtosecond laser (Pharos, Light Conversion, Lithuania) was used in the experiments, featuring a center wavelength of 1030 nm, a pulse duration of 216 fs, a repetition frequency of 10 kHz, and a diameter of 6 mm. To achieve a microstructure with a larger depth-to-diameter ratio, an axial conical lens (taper angle = 175°) was used to transform the Gaussian beam of the femtosecond laser into a Bessel beam. To ensure the precise processing of the ASS, the diameter of the Bessel beam was further minimized to under 3 µm by employing a convex lens (f = 150 mm) and a 40× objective lens, and the Bessel beam length is approximately 200 μm. To achieve a greater depth of ASS after laser processing, a pulse picker was used to generate a pulse train with the same pulse energy but varying numbers of sub-pulses (t_sep_ = 25 ns), as illustrated in Figure 1c. Additionally, burst modes 1 through 8 were sub-pulses of the pulse train, corresponding to pulses numbers 1 through 8, respectively. Commercial ZnS samples (30 mm × 30 mm × 2 mm) were fixed on a 3D motion stage with a motion repeatability of 20 nm. The machining path is shown in Figure 1d.

The ASS surface morphologies were measured by using a scanning electron microscope (SEM, Tescan MIRA), while the profiles were measured by a confocal laser scanning microscope (LCSM, Carl Zeiss, Germany). The infrared transmission spectra of the ASS were obtained using an infrared microscope (FTIR, Thermo Fisher, USA). The wavelength range (2.5 μm to 25 μm) and the incident angle of light (θ = 0°) were specified for infrared transmittance measurements. The wettability of the samples was obtained with an optical contact angle meter (Hacker, China). An infrared detection system, comprising an infrared window, an infrared object, and an infrared camera (FLIR, USA), was assembled to capture infrared images of the ASS.

## 3. Results and Discussion

Figure 1a shows the anti-reflective optical properties of the biological cicada, which inspired the design concept of the ASS. Additionally, the cicada wings are light, and with the array of the nanopillar structure, it has excellent transmission performance and hydrophobic characteristics. Inspired by these biomimetics structures, we fabricated anti-reflective subwavelength structures on a zinc sulfide surface using femtosecond laser burst Bessel beams. As shown in Figure 1b, Bessel beams offer several advantages over Gaussian beams, including an ultra-long depth of focus, high central core intensity, and uniform intensity distribution. However, laser energy significantly attenuates during propagation due to absorption and scattering by the material. The ASS on the ZnS surface is shown in Figure 1e. For the ASSs, the wavelength range of infrared-enhanced transmittance depends mainly on the structural period, while the numerical magnitude of the transmittance depends on the structural depth [32]. In addition, transmittance is closely tied to the microstructural profile and filling factor. A larger filling factor increases the transmittance [30]. The variations in the microstructure profile are primarily attributed to changes in depth. Therefore, in this paper, the depth and period parameters are focused on. Consequently, in order to obtain better transmittance, the ASS parameters need to be optimized by theoretical calculations and simulations before fabrication.

The initial parameters of the ASS can be obtained by the subwavelength diffraction theory [33], and the formula for period (p) is as follows:

(1) $$\frac{p}{\lambda }<\frac{1}{n_{ZnS}+n_{air}}$$

where n_ZnS_, n_air_, and λ represent the refractive indices of zinc sulfide and air and the wavelength of the incident light, respectively. According to Equation (1), ASS can enhance the transmittance of ZnS when the period is less than 3.2 μm, which is due to the fact that ZnS is mainly utilized in the mid-wave infrared broadband range of 2.5–13.5 μm. Furthermore, the anti-reflective structural layer can be considered as ARC [33], according to the effective medium approximation theory. Then, the anti-reflective structural layer depth (h) can be described as follows:

(2) $$h=\frac{\lambda }{4n_{eff}}(2k+1)$$

where n*_eff_* and k are the effective refractive index of the ASS and the constant (k = 0, 1, 2, 3……), respectively. Meanwhile, the transmittance (T) of the ASS layer can be determined using Equation (3) [33]:

(3) $$T=1-\frac{{\left(n_{ZnS}-\frac{n_{eff}^{2}}{n_{ZnS}}\right)}^{2}}{{\left(n_{air}+\frac{n_{eff}^{2}}{n_{ZnS}}\right)}^{2}}$$

It is calculated that the optimal n_eff_ value should be 1.48 to obtain the maximum transmittance (T). Therefore, for the mid-infrared wavelengths, a depth of 1.5 μm is recommended for the array anti-reflective subwavelength structures.

**Figure 2:**
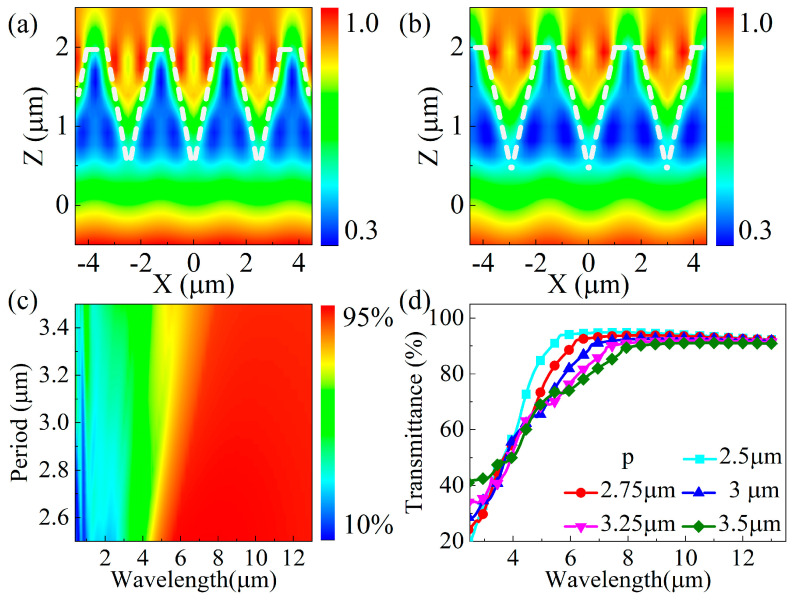
(**a**,**b**) The simulated electric field strength distribution at an incident wavelength of 10 μm for ASS periods of 2.5 μm and 3.0 μm; (**c**) a contour plot of the simulated ASS transmittance versus the incident wavelength, λ, and period, p; (**d**) the simulated transmission spectra of the ASS for wavelengths ranging from 2.5 to 13.5 μm.

In order to research the anti-reflection mechanism of the ASS, simulations of the electric field (light field) intensity distributions were performed. Figure 2a,b show the results for a 1.5 μm deep conical ASS with periods of 2.5 and 3 μm. When the wavelength of the incident light is 10 μm, the light field intensity enhancement region is located in the inner and upper positions of the conical ASS. Additionally, the value of the light field intensity near the interface region on both sides is greater than that at the top location of the conical microhole. The primary reason for this phenomenon is the collision and reflection of the incident beam on the side walls of the anti-reflective structure, caused by the compression of the space within the structure. The smaller the period, the smaller the space inside the structure. The reflection and collision probability of the incident light increases, and the intensity of the light field in the side wall region of the structure is larger. However, when the period increases, the reflection and collision probability of incident light in the middle of the structure decreases, and the intensity of the light field in the middle region becomes smaller. Furthermore, the local light field selective enhancement that occurs in the internal structure can effectively capture the incident IR light and reduce Fresnel reflection from the ZnS surface [27]. Figure 2c shows the transmittance of the ASS with a depth of 1.5 μm at different incident wavelengths, illustrating the selectivity of the period on the transmission wavelength range. In particular, when the wavelength of the incident light and period of the ASS are close to each other, the transmittance value decreases and there is no anti-reflective effect due to the scattering effect, leading to an increase in the higher-order diffraction component. However, as shown in Figure 1d, when the wavelength of the incident light is greater than 8 μm, the small change in period has almost no effect on the transmittance value, which is more than 90%.

**Figure 3:**
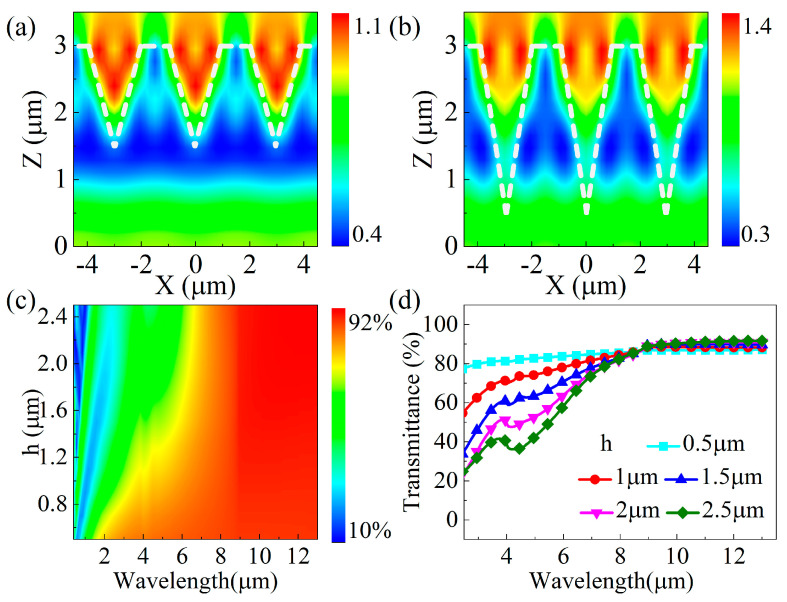
(**a**,**b**) The simulated electric field strength distribution at an incident wavelength of 10 μm for ASS depths of 1.5 μm and 2.5 μm; (**c**) a contour plot of simulated ASS transmittance versus the incident wavelength, λ, and depth, h; (**d**) the simulated transmission spectra of the ASS for wavelengths ranging from 2.5 to 13.5 μm.

The simulated electric field strength distributions for an incident wavelength of 10 μm and ASS depths of 1.5 μm and 2.5 μm are shown in Figure 3a,b. The electric field intensity distributions of the two ASSs at different depths are very different; the electric field strength increases with the depth of the ASS. The extreme value of the electric field strength is 1.0 for the ASS at a depth of 1.5 μm and 1.4 for the ASS at a depth of 2.5 μm. In addition, when the depth increases, the distribution of the electric field intensity remains concentrated in the inner region of the microhole, and the area of the localized light field enhancement region becomes larger. Furthermore, the feature that the light intensity at the interface on both sides is higher than that at the center becomes more and more obvious. This result indicates that the greater the depth of the ASS, the greater the number of collisions and side wall reflections of the incident beam inside the structure. The transmittance spectra in Figure 3c,d show that the transmittance increases from 20% to 85% as the wavelength becomes larger in the range of 2–8 μm. The transmittance of the ASS at 8 μm to 13 μm is about 85% at a depth of 0.5 μm, and the average transmittance is more than 90% when the depth exceeds 1.5 μm. On the one hand, the increase in depth improves the ability of the ASS to capture the incident IR light. On the other hand, the increase in depth results in a smoother effective refractive index gradient in the ASS, which can effectively enhance the transmission by suppressing the surface Fresnel reflection. Therefore, in order to achieve good anti-reflective properties in the ASS on the zinc sulfide surface, the period of the ASS should be limited to less than 3 μm, and the depth of the ASS should be deepened.

**Figure 4:**
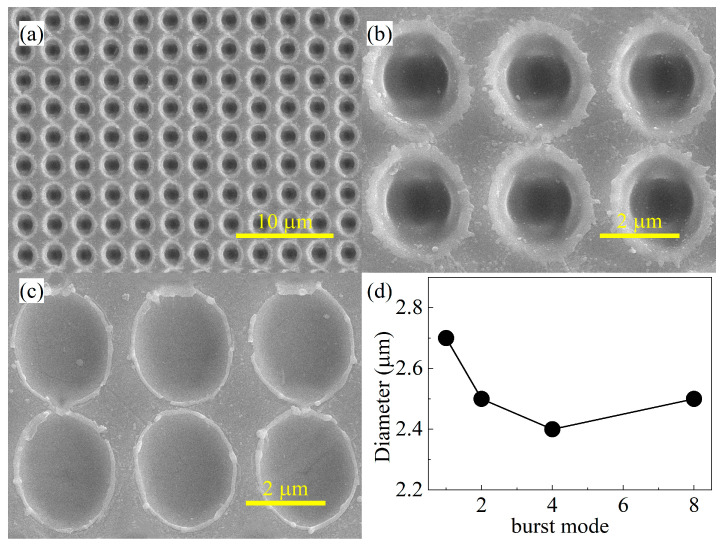
The SEM of the ASS with 6 μJ pulse energy: (**a**,**b**) burst 8; (**c**) burst 1. (**d**) The diameters for different parameters of the ASS.

In order to make the structures fabricated by the femtosecond laser deeper and more efficiently processed, in this paper, the ASS was prepared on the surface of zinc sulfide using a femtosecond laser Bessel beam. Figure 4a shows the scanning electron microscope image (SEM) at a laser pulse energy of 6 μJ. The ASSs prepared using the femtosecond laser have a period of 3 μm and are arranged in a tetragonal shape on the ZnS surface. Comparing the morphology of a microhole prepared under the two parameters of burst 8 b and burst 1c, a clear recast layer appears at the edge of the micropores in the burst 8 mode. The main reason for this result is that the thermal effect of laser ablation is caused by the cumulative effect of multiple sub-pulses. Figure 4c shows the diameters of the ASS prepared at a laser pulse energy of 6 μJ using different laser pulse burst modes. As the burst number increases, the ASS diameter tends to decrease and then increase. Since the total power of the laser is the same, when the energy of the single-pulse laser is distributed into two sub-pulses, the diameter of the laser decreases with decreasing energy, and the diameter of the prepared ASS tends to become smaller. However, with an exponential increase in the number of sub-pulses, the ablation process of a femtosecond laser becomes more and more intense, which ultimately leads to a larger diameter of the ASS.

**Figure 5:**
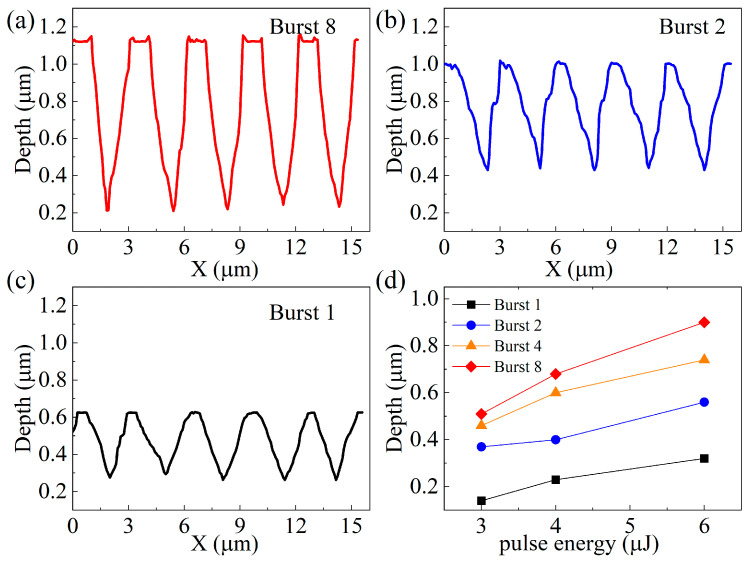
The profiles of the ASS with 6 μJ pulse energy and (**a**) burst 8, (**b**) burst 2, and (**c**) burst 1 mode; (**d**) the profiles of the ASS with different pulse energies and burst modes.

Figure 5a–c show the profile morphology and depth of the ASSs prepared in different burst modes at the 6 μJ pulse energy of the femtosecond Bessel laser. The depth of single-pulse laser processing is 0.32 μm, and the morphology of the structural profile resembles a Gaussian curve. The depths of the ASS are 0.56 μm and 0.9 μm for the parameters of burst 2 and burst 8, respectively. Obviously, increasing the number of burst modes can effectively improve the depth of the ASS. Furthermore, as seen when comparing the structural profiles of the three bursting modes, the structural profiles change from Gaussian to conical as the number of burst modes increases. In addition, correlation studies have found that the conical structure has a higher transmittance than the Gaussian structure [16]. As the number of bursts increases, both the ASS profile and depth improve, leading to further enhancement in IR transmission.

The depth results of the ASS on ZnS for different laser pulse energies and burst modes are shown in Figure 5d. Obviously, the growth trend in ASS depths is different at different laser pulse energies. For the femtosecond laser with 3 μJ pulse energy, the ASS depths change from 0.14 μm to 0.37 μm when burst 1 becomes burst 2, and there is a clear trend in depth increase. However, the increase in the depth of the ASS during burst 8 is not significant. As the pulse energy increases to 4 μJ and 6 μJ, the ASS depths values for burst modes 1, 2, and 4 show a linear increase. This phenomenon can be explained by the temperature scale, where the higher the number of bursts, the smaller the individual sub-pulse energy due to the same total power of the femtosecond laser. When the laser pulse energy is above the material threshold, the number of sub-pulses increases, the cumulative effect of the femtosecond Bessel beam ablation increases, and the depths of the ASS are enhanced. However, lower pulse energies lead to smaller sub-pulse laser ablation efficiencies. Finally, if the sub-pulse energy is less than the material threshold, the cumulative effect of the sub-pulse cannot significantly increase the ASS depth. Meanwhile, laser-machined anti-reflective subwavelength structures are deeply saturated at burst 8 due to reflections from the underside of the structure and plasma generated during processing.

**Figure 6:**
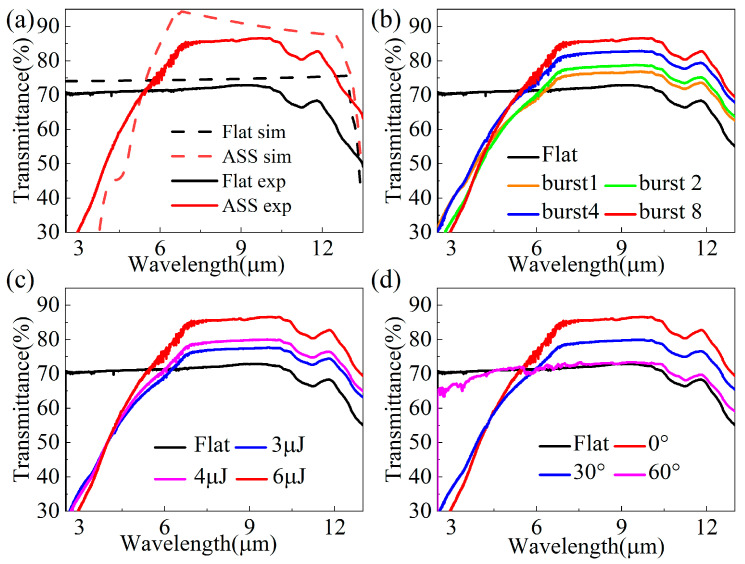
(**a**) Simulated and experimental infrared transmittance of flat ZnS and ASS. Infrared transmittance spectra in (**b**) different burst modes, (**c**) different pulse energies, and (**d**) different incident angles.

The infrared transmission spectra of ZnS prepared with different laser parameters can be clearly seen in Figure 6. In Figure 6a, the IR transmittance in the range of 2.5–12.5 μm for the RCWA simulation and the ASS prepared in this paper can be seen, and the two results are in close agreement. The main reason for the discrepancy is that the simulation process does not take into account the scattering effect caused by the roughness of the ZnS surface. Figure 6b shows the infrared transmission spectra of the ASS for different burst modes at a laser pulse energy of 6μJ. The IR transmittance of the ASS in the wavelength range of 7–10 μm gradually increases with an increasing burst mode, and the corresponding transmittances are 76%, 78%, 82%, and 86%, respectively. Furthermore, the infrared transmission of the ASS improves with an increasing laser pulse energy. In burst 8 mode, the average transmittance in the 5–12 μm range is increased by 10%. Comparing the results in Figure 5 and Figure 6, it can be seen that the evolution patterns of transmittance and the ASS depth with laser parameters are basically the same. This further indicates that utilizing the burst mode can enhance the ASS depth, improve the ASS profile, and effectively enhance the IR transmittance of ZnS. In addition, the IR transmittance of the ZnS surface used to prepare ASS is 73% when the angle between the ZnS sample and the incident beam is 60°, which is still slightly higher than the 72% value of flat ZnS. It is demonstrated that good wide-angle anti-reflective properties can be obtained when the ASSs were prepared on zinc sulfide surfaces.

**Figure 7:**
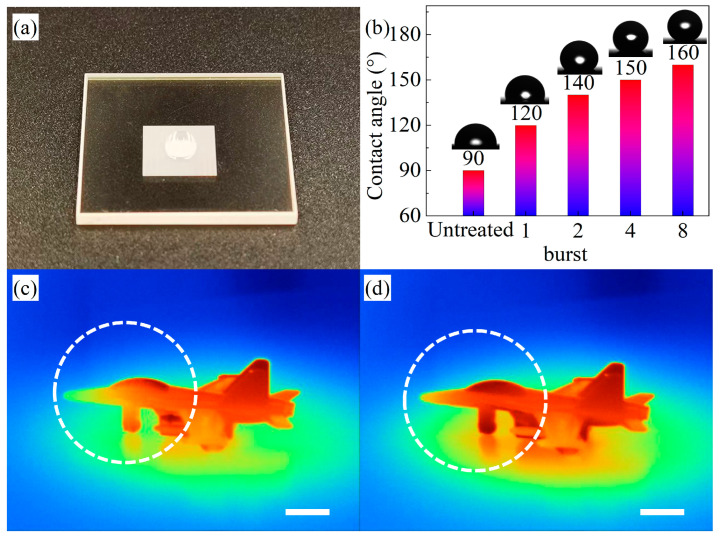
(**a**) A hydrophobic image of the ASS fabricated on the surface of zinc sulfide; (**b**) the wettability of the ASS; infrared thermogram images of flat ZnS (**c**) and ASS (**d**). The white scale bar represents 5 mm.

Additionally, the hydrophobicity of the ASS on the ZnS surfaces was also investigated in this paper, and the contact angles of the laser-processed specimens were tested in different bursting modes. Figure 7a shows the hydrophobicity of the ASS, and Figure 7b shows the detailed results of the contact angles of the ASS. As the burst mode increases from 1 to 8, the contact angle increases to 120°, 140°, 150°, and 160°, respectively. Similar to the evolution pattern of the transmittance, the contact angle also increases with the depth of the ASS. Moreover, as the number of burst modes increases, the diameter of the microstructures tends to decrease. Smaller microstructure diameters allow the liquid droplets to suspend on the microstructures, reducing the solid–liquid contact area and enhancing the hydrophobic performance of the zinc sulfide surface. According to the Wenzel model [34], the increase in depth leads to an increase in the surface roughness coefficient of zinc sulfide, which enhances its hydrophobic properties. Therefore, ZnS windows with ASS surfaces are more suitable for foggy, rainy, and humid environments and have some self-cleaning properties.

In order to better research the anti-reflective effect of the ASS, in this paper, we tested and analyzed the infrared image data of zinc sulfide. It can be seen in Figure 7c,d that the details of the model airplane are clearer, and the outlines are more intelligible. The clarity (IC) grew from 8.5 to 9.6 after the ASS was prepared on the ZnS surface at a temperature of 313 K. The fabrication of anti-reflective subwavelength structures on ZnS window surfaces can enable infrared detection devices to receive more IR signals and possibly promote the application of anti-reflective technology in the IR detection of dim targets.

## 4. Conclusions

In conclusion, the bionic anti-reflective subwavelength structures are fabricated on the ZnS surface by using a femtosecond burst Bessel laser and designed and optimized to eliminate surface Fresnel anti-reflections by the RCWA method. In order to obtain anti-reflective structural parameters, the initial period and depth of the ASS are calculated by using the EMA theory. The anti-reflection mechanism of the ASS is related to the local light field selective enhancement, which can effectively capture the incident infrared light and achieve anti-reflection. Then, the anti-reflective subwavelength structures’ parameters are optimized by RCWA simulations to achieve high transmittance in the mid-infrared range. When the number of bursts increases, the depth of the ASS grows from 0.32 μm to 0.9 μm (bursts 1 to 8), and the structural profiles transition from Gaussian to conical. Additionally, the average transmittance is increased by 10% in the range of 5–12 μm, and the 86% transmittance is achieved in the range of 7–10 μm. In particular, the ASS demonstrates wide-angle anti-reflective properties and hydrophobic properties (contact angle 160°). Finally, the infrared detection measurements show that the ASS can effectively improve the clarity of the infrared image. The proposed fabrication method for the preparation of an ASS can provide insights for civil and military infrared detection.

## Data Availability

The raw data supporting the conclusions of this article will be made available by the authors without undue reservation.
